# Shoulder reduction on the scene: current practice and outcome of the Bavarian Mountain Rescue Service—a prospective observational study

**DOI:** 10.1038/s41598-023-47464-3

**Published:** 2023-11-18

**Authors:** Simon Woyke, Johannes Pawlak, Tomas Dal Cappello, Georg Schultheiss, Herbert Mayer, Ulrike Witt, Giacomo Strapazzon, Hermann Brugger, Matthias Jacob

**Affiliations:** 1 Bavarian Mountain Rescue Service, Am Sportpark 6, 83646 Bad Tölz, Germany; 2 Department of Anaesthesiology and Intensive Care Medicine, Medical University of Innsbruck, Innsbruck, Austria; 3 Institute of Mountain Emergency Medicine, Eurac Research, Bolzano, Italy; 4 Landesamt für Gesundheit und Lebensmittelsicherheit, Task Force Infectiology, Munich, Germany; 5 Department of Anaesthesiology and Intensive Care Medicine, RoMed Clinic Bad Aibling, Bad Aibling, Germany; 6 Department of Traumatology and Orthopedic Surgery, Clinic Immenstadt, Immenstadt, Germany; 7 Emergency Department, Krankenhaus Agatharied, Hausham, Germany; 8 International Commission for Mountain Emergency Medicine (ICAR MedCom), Zürich, Switzerland; 9 Department of Anaesthesiology, Intensive Care and Pain Medicine, Barmherzige Brüder Klinikum St. Elisabeth Straubing GmbH, Straubing, Germany; 10 Faculty of Medicine, Ludwig Maximilian University, Munich, Germany

**Keywords:** Risk factors, Signs and symptoms, Clinical trial design, Musculoskeletal system

## Abstract

Out-of-hospital reduction of shoulder dislocations using the Campell method is recommended by the International Commission for Alpine Rescue and applied in the Bavarian Mountain Rescue Service (Bergwacht Bayern, BWB) protocols. This prospective observational study includes patients out-of-hospital with suspected shoulder dislocation and treated and evacuated by the BWB. Data were systematically collected using three questionnaires: one completed on-site by the rescuer, the second in hospital by the physician and the third within 28 (8–143) days after the accident by the patient. The suspected diagnosis of shoulder dislocation was confirmed in hospital in 37 (84%) of 44 cases. Concomitant injuries in other body regions were found in eight (16%) of 49 cases and were associated with incorrect diagnosis (*p* = 0.002). Younger age (*p* = 0.043) and first shoulder dislocation event (*p* = 0.038) were associated with a higher success rate for reduction attempts. Out-of-hospital reduction of shoulder dislocations leads to significant pain relief and no poorer long-term outcome. Signs that are associated with successful out-of-hospital reduction (younger age and first event), but also those that are associated with incorrect diagnosis (concomitant injuries) should be considered before trying to reduce shoulder dislocation on site. The considerable rate of incorrect first diagnosis on site should give rise to an intensive discussion around teaching and training for this intervention.

*Trial registration*: This study is registered with the German Registry for Clinical Trials (DRKS00023377).

## Introduction

Shoulder dislocation often occurs in skiing accidents, mountain bike accidents or in canyoning incidents^1–3^. Shoulder dislocations are a rare event by comparison with other emergencies^4,5^. However, compared to dislocations of other joints, the shoulder dislocation is, by far, the most common^6^. This injury is painful and immediate successful reduction, representing the causal and effective pain therapy, not only markedly facilitates evacuation. Early reduction might also reduce the incidence of secondary damage like neurologic or vascular dysfunction and long-term complications like paresis and recurrent shoulder dislocations^5,7^. In contrast, delaying reduction to get an X-ray enables accompanying injuries around the glenohumeral joint to be recognized or excluded and, thus, the avoidance of irreversible damage to the joint by a “blind” or, even more, a not indicated reduction procedure for a fractured limb. Retrospective studies addressing the benefit of an early reduction reported an older age (i.e. age > 40 years), a first episode of dislocation and the mechanism of injury as predictors for fracture dislocations potentially complicating a reduction procedure^8,9^.

**Figure 1:**
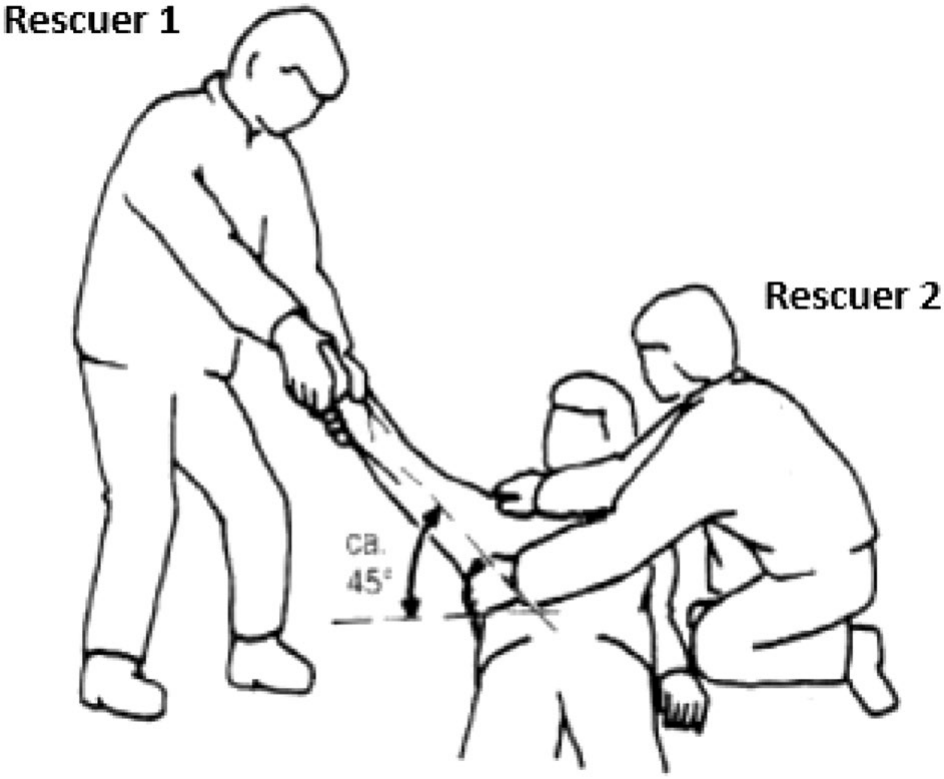
The Campell method with two rescuers for reduction of shoulder dislocation, adapted from Forster and Zafren^10^.

Rescuers working with the Bavarian Mountain Rescue Service (Bergwacht Bayern, BWB) are systematically taught and trained to diagnose and, if indicated, to reduce shoulder dislocations on-site, namely according to the recommendation of the International Commission for Alpine Rescue, Commission for Mountain Emergency Medicine (ICAR MedCom), particularly when pain is intense or there is motor or sensory dysfunction or impaired perfusion in the affected arm^10^. Not only emergency physicians, but also other mountain rescue team members with basic medical training are encouraged to reduce shoulder dislocations on-site before evacuation if no physician is available on the scene within a reasonable time. This intervention is taught and usually performed using the Campell method as recommended by ICAR MedCom^10–12^. The Campell method calls for one rescuer to exert continuous, gentle and longitudinal traction in order to abduct the affected arm up to 90° at a 45° angle to the ground, while a second rescuer stabilizes the patient’s trunk in order to keep him/her in a supine position (Fig. 1)^10^. Another popular method is FARES (FAst, REliable and Safe). This is also based on gentle longitudinal traction of the affected arm, and abduction of the arm while oscillating^13^, but is not regularly trained by BWB. However, international guidelines and recommendations on out-of-hospital management of shoulder dislocations are indeterminate^14^. Other emergency medical services (EMS), especially those that do not usually operate in mountainous environments (but also EMS operating in mountain areas with time-consuming technical rescue procedures) take a more conservative approach. They avoid reducing shoulder dislocations on-site and perform reductions only in hospital after taking an X-ray to exclude fractures around the joint.

On-site shoulder reduction performed by physicians, ski patrollers or first responders shows quite good success rates, with complications caused by the reduction itself being relatively rare^4,15,16^. Recently, Mulvey and colleagues evaluated the success rates of first responding ski patrollers performing reduction of anterior shoulder dislocations on-site mainly using the Cunningham method. Unfortunately, important data for sufficient interpretation of the results, like final diagnosis, post-reduction attempt X-rays or complications, were hardly reported^17^. The Campell method, performed by an organized mountain rescue service, together with the final diagnosis and the related outcome, have not yet been addressed.

Our prospective clinical study aims to evaluate the current BWB practice. As out-of-hospital diagnosis is made based solely on clinical signs and symptoms, the definitive clinical diagnosis was one of our special interests.

## Methods

The present study was performed in accordance with the relevant guidelines and regulations. This is a prospective observational study that included patients of any age with suspected traumatic shoulder dislocation (clinical signs and symptoms, e.g., a visibly deformed or out-of-place shoulder, swelling or bruising, intense pain, inability to move the arm), who were treated and evacuated by BWB between October 2020 and March 2022. Data were systematically collected in three questionnaires: a first one was completed by the rescuer on-site and contained questions around initial signs and symptoms, concomitant injuries in other body regions, pain intensity before and after reduction attempt, time intervals, and, finally, reduction method. The questionnaire was sent to the study team in a sealed envelope. Two to four weeks after the accident, an information letter with the informed consent disclosure form and a follow-up questionnaire was sent to the patient. This questionnaire included questions concerning current pain intensity after weeks and possible neurological complications or remaining deficits. If informed consent was given by the patient, the first out-of-hospital envelope was then opened, otherwise the case was excluded from the study and the sealed envelope was destroyed. A third questionnaire containing information about the final diagnosis, X-ray findings, complications and the in-hospital reduction method, if not already reduced on-site, was then completed with the help of the staff of the admitting hospital.

Throughout the study, the numeric rating scale (NRS) was used to assess pain intensity at the respective moment on a 0–10 scale, with zero meaning “no pain” and 10 meaning “the worst pain imaginable”^18^. NRS was assessed before and after a reduction attempt.

The software program SPSS, version 27 (IBM Corp., Armonk, NY, USA), was used for statistical analysis. Comparison of frequencies between three groups was performed by means of Pearson’s chi-squared test, while comparison of frequencies between two groups was performed with Fisher’s exact test. Comparison of continuous variables between two groups was performed by means of independent samples *t* test or Mann–Whitney U test, as appropriate. Pain level before and after the reduction attempt was compared with a paired sample *t* test. Logistic regression was performed to analyze the effect of an out-of-hospital reduction attempt and the time from accident to completing the questionnaire on motor dysfunction in follow-up data. Tests were two-sided and *p* < 0.05 was considered statistically significant. Values of normally distributed variables are reported as mean ± standard deviation, while variables of not normally distributed variables are quantified by the median (range). Frequencies are reported as percentages.

### Ethics approval and consent to participate

The present study was approved by the Ethics Committee of the Faculty of Medicine of Ludwig Maximilian University Munich (Project 20-0504). All included patients gave written informed consent.

## Results

**Figure 2:**
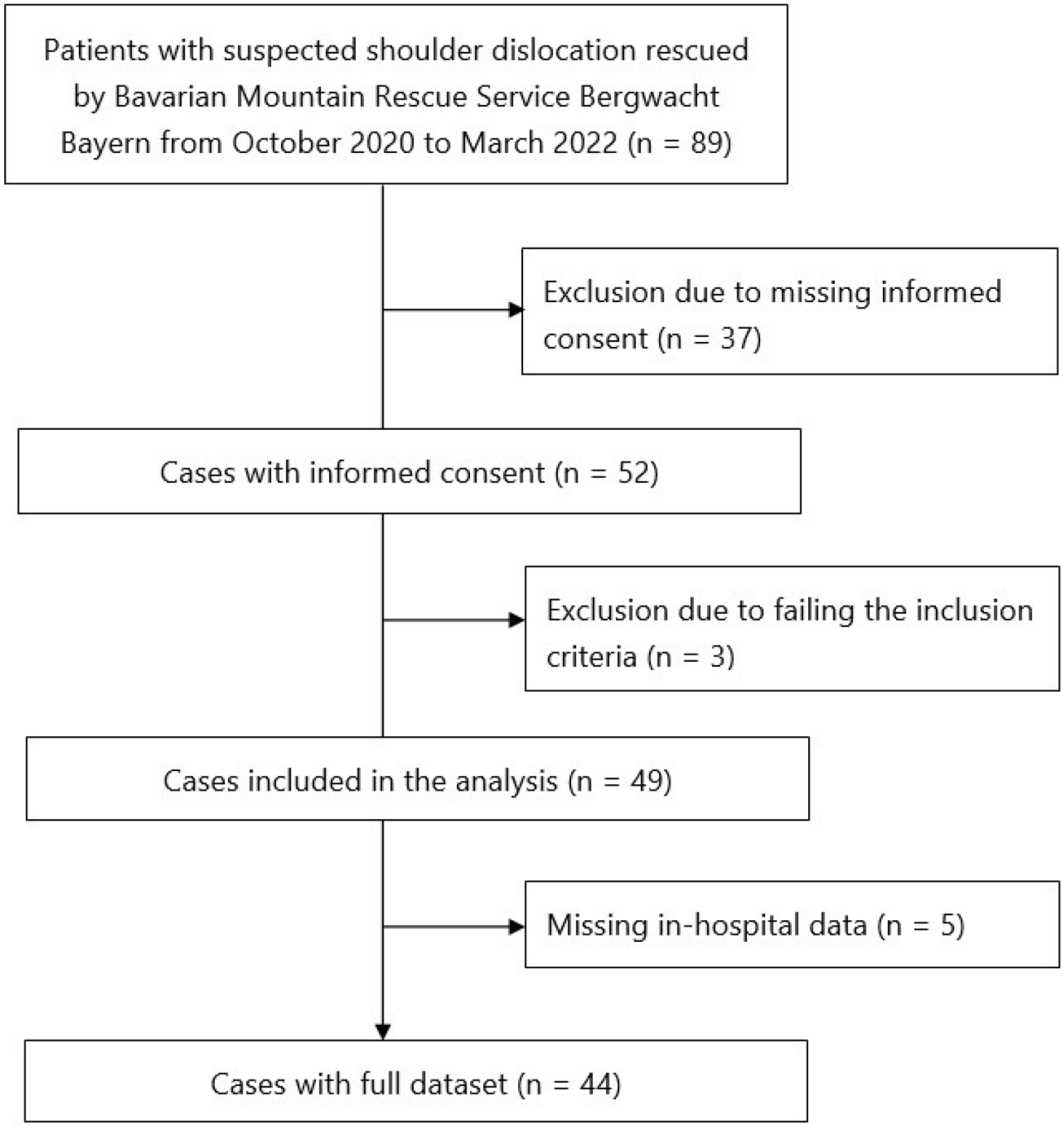
STROBE flowchart for “Shoulder Reduction on the Scene—Current Practice and Outcome of the Bavarian Mountain Rescue Service.” Thirty-seven patients had to be excluded because they failed to return the informed consent form. Three patients with suspicion of fracture failed to meet the inclusion criterion of suspected shoulder dislocation and were excluded.

Eighty-nine patients with traumatic shoulder dislocation were identified; 49 of them (age 42 ± 18 years) were recruited and 44 of them had a complete dataset (out-of-hospital, in-hospital and follow-up data), while in five cases the in-hospital data were missing (see the STROBE flowchart in Fig. 2). Three cases were excluded, as they were suspected to suffer fracture and the inclusion criterion “suspected shoulder dislocation” was not met.

Of the 44 patients 12 (27%) with a complete dataset were female and 32 (73%) were male. Most accidents occurred while skiing [27 (55%)], followed by trekking [6 (12%)], snowboarding [5 (10%)], and mountain biking/ski touring [3 (6%) each]. One (2%) accident occurred while e-biking, paragliding, or snow-biking each. The majority of accidents occurred on ski slopes [32 (65%)] or on streets or paths [6 (12%)], with only one (2%) accident being in an unsafe area (rockfall).

As shown in Table 1, most patients suffered from isolated shoulder dislocation, which was the first event in the majority of patients. Table 1 shows pain levels at first contact as well as motor, sensory dysfunction or impaired perfusion at first contact.

**Table 1:** Cases of shoulder dislocation classified based on possible concomitant injury, first shoulder dislocation event, and weather permitting helicopter rescue. Pain levels at first contact, according to the numeric rating scale (NRS). Appearance of sensory or motor dysfunction (due to pain or limitation in range of motion) or impaired perfusion at first contact. Data refer to all out-of-hospital data (n = 49). One patient can suffer from more types of dysfunction.

	Yes		No	Unknown
Concomitant injury in other body regions, n (%)	8 (16%)		34 (70%)	7 (14%)
First-event, n (%)	37 (76%)		10 (20%)	2 (4%)
Weather permits helicopter rescue, n (%)	46 (94%)		3 (6%)	0 (0%)
	NRS 0–3	NRS 4–6	NRS 7–10	Unknown
Pain level at first contact	7 (14%)	9 (19%)	32 (65%)	1 (2%)
No dysfunction	Motor dysfunction	Sensory dysfunction	Impaired perfusion	Unknown
30 (61%)	3 (6%)	12 (24%)	5 (10%)	2 (4%)

### Out-of-hospital reduction attempts

In 34 (69%) of the 49 cases included in the analysis reduction was attempted out-of-hospital. In most cases the operator of the reduction attempt was an emergency physician [18 (53%)] or mountain rescue paramedic [10 (29%)]. In six cases (18%) it was a mountain rescue team member with basic medical training. Median time from accident to first reduction attempt was 15 (3–60) minutes, duration of reduction attempts was 5 (1–15) minutes. In 18 (53%) cases the Campell method was used, in four (12%) cases the FARES method, in eight (23%) cases another method (Cunningham, Spaso and scapular manipulation technique) and in four cases (12%) the applied technique was not specified. In one case two different methods were used, both of which failed. Judging from clinical signs and symptoms, the operator considered the reduction attempts to be successful in 27 (79%) cases, and in seven (21%) not successful. In the majority of cases (71%) the reduction was successful on the first attempt; only three cases required a second attempt for success. No further reduction attempt led to a successful result. When reduction was not successful, in four cases only one attempt was made, while in one case each two attempts, three attempts or five attempts were undertaken. In two cases with incorrect suspicion of shoulder dislocation (i.e., other injury around the glenohumeral joint) out-of-hospital reduction was attempted, and reduction was deemed successful in one of them. Before and during the reduction attempt, 17 (50%) patients did not receive analgesic drugs, 17 (50%) patients received analgesic treatment (in seven patients fentanyl, in five midazolam and ketamine, in one fentanyl and midazolam and ketamine and in four patients the administered drugs were not reported). Pain levels immediately following the reduction attempt were significantly lower [NRS 0–3 in 27 (79%), NRS 7–10 in 2 (6%), unknown in 5 (15%) cases] than the level before the attempt (pain levels before reduction attempt in Table 1) (*p* < 0.001). At first contact by the rescue team a motor, sensory or perfusion dysfunction was apparent in almost all cases. These clinical signs were markedly reduced immediately after the reduction attempt (except in one patient). In none of the cases was a deterioration of the clinical signs reported after the reduction attempt. Sixteen (33%) patients were transported to hospital by helicopter and 32 (65%) terrestrially.

### Analysis of in-hospital data

The following analysis refers to the 44 cases with a full dataset, including in-hospital data. In 37 (84%) patients the out-of-hospital diagnosis of shoulder dislocation was correct according to X-ray findings. For nonmedical mountain rescuers and physicians, the pre-hospital diagnosis was always correct, while for paramedics the diagnosis of shoulder dislocation was correct in eight (80%) of ten cases. When no concomitant injury in other body regions was suspected on-site, the diagnosis of shoulder dislocation was more often correct than when another injury was suspected (93% vs. 38%, *p* = 0.002, n = 38). When the on-site diagnosis of shoulder dislocation was incorrect, other injuries around the glenohumeral joint were present (multiple fractures in the shoulder joint in one, multiple fractures in the acromioclavicular joint in one, luxation fracture of the humerus head in two, fracture in the shoulder joint not further specified in one, humerus fracture in one and injury of the acromioclavicular joint in one patient).

### Follow-up data

Follow-up questionnaires were completed by the patients (n = 49) within 28 (8–143) days after the accident. Successful reduction on-site was not associated with pain (*p* = 1, n = 31), sensory loss (*p* = 1, n = 31) or motor function loss (*p* = 0.394, n = 31) in follow-up data. An out-of-hospital reduction attempt in general was associated with less motor dysfunction (*p* = 0.042) in follow-up data.

### Characteristics of successful out-of-hospital reduction attempts

To exclude cases with false successful reduction, the following paragraphs refer solely to those cases with correct diagnosis (n = 37), as confirmed by in-hospital data. Patients with successful reduction were younger than were those with unsuccessful out-of-hospital reduction attempts (33 ± 14 years vs. 47 ± 17 years, *p* = 0.043, n = 29). If the dislocation was the patient’s first such event, reduction was associated with a higher success rate (90% vs. 50%, *p* = 0.038, n = 28). The operator’s formal qualification did not have an impact on the success of the out-of-hospital reduction (medical doctor vs. paramedic vs. mountain rescuer, *p* = 0.182, n = 29). The chosen reduction method had no effect on success rate (Campell vs. FARES vs. other, *p* = 0.864, n = 27). Success of out-of-hospital reduction was affected neither by the initial pain level (*p* = 0.296, n = 28) nor by sensory or motor loss or clinically impaired perfusion (*p* = 0.607, n = 29). Patients who received analgesics were not more likely to have a successful reduction in the field (73% vs. 85%, *p* = 0.655, n = 28). The success rate of out-of-hospital reduction was not associated with complications such as Hill-Sachs or Bankart lesions (*p* = 0.175, n = 22).

Patients with a history of previous dislocation were not more likely to undergo attempted out-of-hospital reduction (89% vs. 77%, *p* = 0.648, n = 35). In 14 of 34 cases with attempted out-of-hospital reduction patients suffered no vascular, sensory, or motor impairment. All of these 14 patients reported moderate to severe pain levels (3 cases NRS 4–6, 11 cases NRS 7–10). Thus, there was no case where out-of-hospital reduction was attempted in a patient with no dysfunction or with low pain levels. When out-of-hospital reduction was attempted, pain level decreased by 5.3 ± 2.7 and there was no case with an increase in pain level after reduction. Only in one patient with five consecutive unsuccessful reduction attempts did the pain level remain the same. Time from accident to hospital did not differ depending on whether out-of-hospital reduction was attempted or not, but comparison was close to statistical significance [75 (44–172) min without attempt vs. 102 (48–330) min with attempt, *p* = 0.080, n = 25].

## Discussion

This study shows that younger age and first shoulder dislocation event are associated with a successful out-of-hospital reduction attempt. The diagnosis of shoulder dislocation based on clinical signs and symptoms was wrong in every sixth case, and the presence of concomitant injuries in other body regions increased the likeliness of incorrect preclinical assessment. Adherence to current recommendations and the BWB syllabus on how to treat patients with shoulder dislocations in rough terrain was high as all patients that were subject to an out-of-hospital reduction attempt suffered severe pain or motor, sensory or vascular dysfunction. Out-of-hospital reduction attempts had no negative impact on long-term outcome like persistent pain, motor or sensory dysfunction. Motor dysfunction was less frequent in those patients with out-of-hospital reduction attempts. The study population included both young and old patients, with the majority being male. Different risk behavior might play an important role^19^. Most of the included accidents were located on ski slopes and happened while skiing or snowboarding.

The first shoulder dislocation event combined with an older age of the victim was reported to be associated with fractures around the glenohumeral joint^9^. In our study, success of out-of-hospital reduction attempts was associated with younger patient age, in keeping with the literature^9^, and with a first shoulder dislocation event. The latter appears to be counterintuitive, as one might expect reduction to be easier if the patient already had several previous dislocations. However, we cannot clearly explain this finding. One attempt at explanation might be that patients with recurrent shoulder dislocations usually reduce themselves immediately after the dislocation so there is no contact with the rescue system. Those that fail at self-reduction due to complicated re-dislocation might be overrepresented in this study. In our data there is no indication that reduction was attempted more often in patients with recurrent shoulder dislocations compared to first event.

Our study revealed a higher incidence of suspected concomitant injuries in those with incorrect diagnosis. Similarly, Emond and colleagues identified the mechanism of injury as the third risk factor for associated fractures^9^. The appearance of concomitant injuries in other body regions might indicate a higher energy or more powerful mechanism of injury and enhance osseous injury/ fractures. Thus, we recommend re-evaluating the diagnosis of shoulder dislocation in such patients and strongly considering differential diagnoses and even more caution before attempting an out-of-hospital reduction. In 16% of the cases with full datasets the out-of-hospital diagnosis of shoulder dislocation was definitely incorrect according to X-ray findings, the real diagnoses being, e.g., fracture of the humerus or the acromioclavicular joint. Notably, in some of these cases the rescuer was so confident that she/he attempted an out-of-hospital reduction, with the rescuer convinced that she/he ultimately successfully reduced the presumed shoulder dislocation. This is interesting because it indicates that studies reporting high success rates for out-of-hospital reduction without reporting the final diagnosis might overlook and misinterpret those cases and, therefore, should be taken with a grain of salt^17^. Moreover, our results highlight how the diagnosis of shoulder dislocation based solely on clinical signs and symptoms is difficult to establish and needs proper education and training. Most of the rescuers in our study, who misinterpreted and falsely assumed that a shoulder was dislocated, were paramedics. Shoulder dislocations may well be overrepresented in professional training courses with regard to other, more common shoulder or humerus injuries, thus leading to a biased focus in clinical examination. During a reduction attempt, the rescuer should carefully consider pain exacerbation or crepitation in the glenohumeral joint and if there is any doubt he/she should discontinue the reduction attempt.

In most of the cases with the out-of-hospital diagnosis of “shoulder dislocation” a reduction was attempted. In all cases of out-of-hospital reduction attempts in our study, impairment of motor, sensory or vascular function or severe pain justified the out-of-hospital reduction according to the present recommendations^10^. In more than half of the included cases the method recommended by ICAR MedCom and favored and trained by BWB (Campell method) was applied. Impairment of motor, sensory or vascular function was apparent before the reduction attempt in more than one-third of all patients, with the sensory function being particularly at risk. Following successful reduction, these dysfunctions disappeared in all cases documented here. Only in one case was a sensory dysfunction documented after an unsuccessful reduction attempt. The follow-up data showed no correlation between success of out-of-hospital reduction and long-term motor, sensory or vascular dysfunction. However, the discrimination of motor dysfunction in comparison to a functio laesa due to persistent pain might be difficult for some patients. Nevertheless, we found no sign that an out-of-hospital reduction attempt might have negative effects on long-term outcome measures like persistent pain, motor or sensory dysfunction. Conversely, the performance of an out-of-hospital reduction attempt was associated with better long-term motor function. Our data indicate that the widespread fear associated with an out-of-hospital shoulder reduction attempt might be overstated if the differential diagnosis and predictors for concomitant injuries around the glenohumeral joint are appropriately considered.

Success of out-of-hospital reduction was not associated with the operator’s formal qualification, pain level or the appearance of motor, sensory or vascular loss, or reduction method. The presence of Hill-Sachs or Bankart lesions was not associated with the success of an out-of-hospital reduction. To our knowledge, this is the first systematic trial investigating the Campell method. Unfortunately, due to the observational study design and small sample size in this study, a rigorous evaluation and comparison with other reduction techniques are not possible. Successful shoulder reduction led in all cases to significant pain relief. Whether an accompanying pain treatment or the successful reduction itself led to this effect cannot be determined from our data. However, markedly and sustainably increased evacuation comfort should mainly be a consequence of a successful reduction. Unfortunately, in three of the six patients with correct diagnosis but unsuccessful reduction, data concerning the pain level following the reduction attempt are missing. Despite considerable high initial pain levels, analgesics were not administered to half of the patients. As the administration of pain medication in Germany is restricted to physicians, this observation might reflect the fact that in only half of the cases physicians were available on the scene in our study. This could obviously be optimized in future, as administration of analgesics even without adequate monitoring was shown to be safe in helicopter emergency medical services^19^.

The median duration of reduction attempts in our study was only a few minutes, while the range was particularly large. Time from accident to start of the reduction procedure also varied over a wide range. There is an indication that time from accident to arrival at the hospital—including time for rescuers to arrive at the accident site, initial treatment on-site and transportation—was longer in those cases with out-of-hospital reduction attempts. Reduction attempts seem to have prolonged duration of initial treatment on-site, with no relevant reduction of transportation time. As one might suspect faster transportation times in patients with successfully reduced dislocations, this might implicate easy transportation options, e.g. on ski slopes. Duration of the out-of-hospital reduction should therefore probably be considered and carefully weighed against evacuation and transport time to definitive care. Point-of-care ultrasound is a reliable method in trained hands to detect shoulder dislocation in either an ambulance or outpatient department. However, environmental conditions like bright light and cold temperature conditions on a ski slope might limit its diagnostic value. In this study ultrasound was not used as a diagnostic tool.

Even though dislocation of the glenohumeral joint is a common injury, the training of mountain rescuers should also focus on other types of injuries in the shoulder region and highlight the need for a rigorous clinical examination on-site with critical consideration of differential diagnoses. Signs that are associated with successful reduction (younger age, first event) and incorrect diagnosis (concomitant injuries in other body regions) should be included in education programs. Bavarian Mountain Rescuers treat patients in accordance with the current ICAR MedCom guideline. Appropriate training of (differential) diagnosis and reduction techniques is needed to enable also non-medical rescuers to reliably balance individual competence, situational importance, expected success and potential damage before attempting to reduce a dislocated shoulder. Particularly in situations where transportation is complicated because the affected arm is fixed in an abducted position and long transportation times are expected, early on-site reduction of a shoulder dislocation might be of benefit. It is encouraging that there was no sign of additional damage caused by a reduction attempt while pain levels were diminished after successful reduction.

## Limitations

Unfortunately, the drop-out rate in our study was high, especially as a result of missing informed consent because patients failed to respond. Furthermore, in five cases clinical data were not able to be collected as the responsible hospital was not compliant despite patients having given their consent. The small sample size and the particular region in which the study took place might limit transferability to other systems in other countries. Due to the observational study design, a selection bias cannot be entirely excluded. Follow-up questionnaires were completed between day 8 and day 143 after the accident by the patient her-/himself, and a differentiation between real motor dysfunction and motor limitation due to pain might be difficult for non-medically trained patients. Furthermore, recent surgery for the shoulder injury prior to completion of the questionnaire might have confounded follow-up pain levels. Compliance of professional ski patrollers to include patients in this study might be greater because such injuries are encountered by them with greater frequency than by other mountain rescuers, indicating a possible selection bias favoring skiing and snowboarding accidents over other sports. If the X-ray showed a different fracture in the shoulder or additional imaging clearly revealed a diagnosis other than the suspected shoulder dislocation, this case was considered to be an incorrect out-of-hospital diagnosis. However, in those cases with successful out-of-hospital reduction the X-ray in most cases cannot prove the diagnosis, but if the diagnosis of “status post” shoulder dislocation was confirmed by the admitting hospital, this was considered a case with correct diagnosis. Due to the observational study design a clear cause-and-effect relationship for the factors associated with a successful reduction attempt cannot be proven.

## Conclusion

With correct diagnosis out-of-hospital reduction of a shoulder dislocation can be safe and can lead to immediate and significant reduction of pain severity. Younger age and first shoulder dislocation event are associated with successful out-of-hospital reduction. By contrast, the appearance of concomitant injuries in other body regions is an indicator for fractures involving the glenohumeral joint and differential diagnoses should be rigorously considered before a reduction attempt.

## Data Availability

The datasets used and/or analyzed during the current study are available from the corresponding author on reasonable request.

## References

[1] Kocher, M. S. & Feagin, J. A. Shoulder injuries during alpine skiing. *Am. J. Sports Med.* **24**(5), 665–669 (1996). 8883689 10.1177/036354659602400517

[2] Aleman, K. B. & Meyers, M. C. Mountain biking injuries in children and adolescents. *Sports Med.* **40**(1), 77–90 (2010). 20020788 10.2165/11319640-000000000-00000

[3] Strapazzon, G. *et al.* International commission for mountain emergency medicine consensus guidelines for on-site management and transport of patients in canyoning incidents. *Wilderness Environ. Med.* **29**(2), 252–265 (2018). 29422373 10.1016/j.wem.2017.12.002

[4] Helfen, T., Ockert, B., Pozder, P., Regauer, M. & Haasters, F. Management of prehospital shoulder dislocation: Feasibility and need of reduction. *Eur. J. Trauma Emerg. Surg.* **42**(3), 357–362 (2016). 26156391 10.1007/s00068-015-0545-5

[5] Beeson, M. S. Complications of shoulder dislocation. *Am. J. Emerg. Med.* **17**(3), 288–295 (1999). 10337892 10.1016/s0735-6757(99)90127-4

[6] Hovelius, L. Incidence of shoulder dislocation in Sweden. *Clin. Orthop. Relat. Res.* **166**, 127–131 (1982). 7083659

[7] Pasila, M., Jaroma, H., Kiviluoto, O. & Sundholm, A. Early complications of primary shoulder dislocations. *Acta Orthop. Scand.* **49**(3), 260–263 (1978). 685670 10.3109/17453677809005762

[8] Orloski, J., Eskin, B., Allegra, P. C. & Allegra, J. R. Do all patients with shoulder dislocations need prereduction X-rays?. *Am. J. Emerg. Med.* **29**(6), 609–612 (2011). 20825841 10.1016/j.ajem.2010.01.005

[9] Emond, M., Le Sage, N., Lavoie, A. & Rochette, L. Clinical factors predicting fractures associated with an anterior shoulder dislocation. *Acad. Emerg. Med.* **11**(8), 853–858 (2004). 15289192 10.1111/j.1553-2712.2004.tb00768.x

[10] Forster, H. & Zafren, K. *Alpine Emergency Medicine Commission—Recommendation 0009—Treatment of Shoulder Dislocations*. Accessed on 27 April 2023. https://www.alpine-rescue.org/articles/49--treatment-of-shoulder-dislocations.

[11] C K. Alpine Traumatologie. In *Alpin- und Höhenmedizin* (ed. Berghold, F) (Springer, Wien, 2015).

[12] Freudig, T. M. A. *Bergrettung. Lehrbuch der Bergwacht Bayern* (1995).

[13] Chamseddine, A. H. *et al.* FARES method for reduction without medication of first episode of traumatic anterior shoulder dislocation. *Int. Orthop.* **43**(5), 1165–1170 (2019). 30159802 10.1007/s00264-018-4131-4

[14] Heitmann, M., Frosch, K.-H. & Wittner, B. *DGU Leitlinie 012-13 Posttraumatische Schulterinstabilität*, 32 (AWMF, 2019).

[15] Ditty, J., Chisholm, D., Davis, S. M. & Estelle-Schmidt, M. Safety and efficacy of attempts to reduce shoulder dislocations by non-medical personnel in the wilderness setting. *Wilderness Environ. Med.* **21**(4), 357–61.e2 (2010). 21168791 10.1016/j.wem.2010.06.010

[16] Bokor-Billmann, T. *et al.* Reduction of acute shoulder dislocations in a remote environment: A prospective multicenter observational study. *Wilderness Environ. Med.* **26**(3), 395–400 (2015). 25823603 10.1016/j.wem.2014.12.027

[17] Mulvey, J. M., Carson, I. N. & Palmer, K. A. Closed reduction of anterior shoulder dislocations performed by ski patrollers in the alpine prehospital environment: A retrospective review demonstrating efficacy in a Canadian ski resort. *Wilderness Environ. Med.* **32**(4), 441–449 (2021). 34635430 10.1016/j.wem.2021.07.007

[18] Karcioglu, O., Topacoglu, H. & Dikme, O. A systematic review of the pain scales in adults: Which to use?. *Am. J. Emerg. Med.* **36**(4), 707–714 (2018). 29321111 10.1016/j.ajem.2018.01.008

[19] Rugg, C., Woyke, S., Voelckel, W., Paal, P. & Ströhle, M. Analgesia in adult trauma patients in physician-staffed Austrian helicopter rescue: A 12-year registry analysis. *Scand. J. Trauma Resusc. Emerg. Med.* **29**(1), 28 (2021). 33526048 10.1186/s13049-021-00839-9 PMC7852148

